# Muscle Activation and Mobility After Robotic Total Knee Arthroplasty: Insights from Early Postoperative Recovery

**DOI:** 10.3390/jcm14093150

**Published:** 2025-05-01

**Authors:** Fernando García-Sanz, Carlos Romero-Morales, Rocío Espejo-Carrizo, Julio Caballero-López, Daniel Sánchez-Clemente, María Bravo-Aguilar, Daniel López-López, Jorge Hugo Villafañe, Sergio L. Jiménez-Saiz, Ángel González-de-la-Flor

**Affiliations:** 1 Department of Physiotherapy, Faculty of Medicine, Health and Sports, European University of Madrid, Villaviciosa de Odón, 28670 Madrid, Spain; fernando.garcia@clinicacemtro.com (F.G.-S.); julio.caballero@universidadeuropea.es (J.C.-L.); maria.bravo@universidadeuropea.es (M.B.-A.); jorge.villafane@universidadeuropea.es (J.H.V.); angel.gonzalez@universidadeuropea.es (Á.G.-d.-l.-F.); 2 Clínica CEMTRO, 28034 Madrid, Spain; daniel.sanchez@clinicacemtro.com; 3 Stryker Orthopaedics, 28108 Madrid, Spain; rocio.espejo@striker.com; 4 Research, Health and Podiatry Group, Department of Health Sciences, Faculty of Nursing and Podiatry, Industrial Campus of Ferrol, Universidade da Coruña, 15403 Ferrol, Spain; daniellopez@udc.es; 5 Sport Sciences Research Centre, Faculty of Education & Sport Sciences and Interdisciplinary Studies, Universidad Rey Juan Carlos, 28943 Fuenlabrada, Spain; sergio.jimenez.saiz@urjc.es

**Keywords:** robot-assisted total knee arthroplasty, quadricep activation, range of motion, surface electromyography, rehabilitation, sex differences

## Abstract

**Background**: Robot-assisted total knee arthroplasty (TKA) has gained attention for its ability to improve surgical precision, optimize component alignment, and potentially enhance functional outcomes. Despite these advantages, early postoperative deficits, particularly in quadricep activation and the range of motion (ROM), remain common and can delay recovery. The objective of this study was to investigate early postoperative differences in quadricep muscle activation and the ROM between the operated and non-operated sides following robot-assisted TKA. **Methods**: A total of 101 participants (50 females, 51 males) were included in the study. Surface electromyography (sEMG) was recorded from the vastus lateralis and vastus medialis during an active knee extension test and a 4-m walking test. The ROM was assessed during gait. A linear mixed model was employed with operated side and sex as factors. **Results**: Significant differences were observed in quadricep muscle activation and the ROM between the operated and non-operated sides. During the knee extension test, males exhibited significantly higher vastus lateralis activity on the non-operated side (mean difference = 174 µV, 95% confidence interval (CI) [90, 258], *p* < 0.001) and females showed a similar pattern (mean difference = 238 µV, 95% CI [152, 324], *p* < 0.001). Additionally, vastus medialis activation was significantly higher on the non-operated side for both males (mean difference = 102 µV, 95% CI [34, 169], *p* = 0.003) and females (mean difference = 137 µV, 95% CI [47, 226], *p* = 0.003). During the 4-m walking test, females displayed a significantly reduced sagittal-plane ROM on the operated side (mean difference = 7.691°, *p* = 0.041) whereas no significant ROM differences were found in males (*p* > 0.903). **Conclusions**: Robot-assisted TKA patients exhibit significant early postoperative asymmetries in quadricep activation and the gait ROM, particularly among females.

## 1. Introduction

Knee osteoarthritis (OA) is a major cause of disability worldwide, with a prevalence that significantly increases with age, affecting approximately 10% of men and 13% of women over 60 years old [1]. Total knee arthroplasty (TKA) is a well-established intervention for end-stage knee OA aimed at relieving pain, restoring function, and improving quality of life [2]. With advances in surgical techniques, robot-assisted total knee arthroplasty has emerged as a promising innovation, offering greater precision in component alignment, soft tissue balancing, and surgical outcomes [3,4,5].

The introduction of robot-assisted systems has transformed the surgical procedure of knee arthroplasty by integrating preoperative imaging, intraoperative guidance, and real-time feedback [6]. These systems allow for patient-specific planning, ensuring the optimal placement and alignment of the implant, which is critical for the long-term survival of the implant [7]. Studies have shown that robot-assisted TKA reduces alignment errors and improves component placement compared to conventional techniques [3,8]. Moreover, better alignment has been associated with greater joint stability, which could lead to better functional outcomes [9].

Despite these technological advances, early postoperative recovery remains a challenging phase for patients undergoing TKA [10]. The immediate postoperative period is characterized by pain and deficits in quadriceps strength, the range of motion (ROM), and functional mobility [10,11]. These deficiencies can delay functional recovery, limit patient satisfaction, and increase the risk of complications such as stiffness or falls [10,12]. Although the precision of robot-assisted TKA may contribute to improving outcomes, the extent to which it influences early functional recovery compared to conventional methods is still unclear [13].

A critical aspect of early recovery involves achieving symmetry between the operated and non-operated limbs during functional tasks such as walking and active knee extension [11]. Previous studies have highlighted the importance of limb symmetry for gait efficiency, balance, and overall mobility [14,15,16]. Early postoperative recovery after total knee arthroplasty (TKA) is characterized by significant asymmetry in lower limb strength, with deficits exceeding 50% in the quadricep muscle of the operated limb [15,16]. However, to the authors’ knowledge, no studies had been investigated about the degree of asymmetry in patients after robot-assisted total knee arthroplasty, particularly in the early postoperative period. Most studies had focused on long-term outcomes, leaving a significant gap in understanding the recovery trajectory during the early postoperative weeks [17,18,19]. The 4-week postoperative interval was specifically chosen in this study to capture short-term neuromuscular deficits and asymmetries, a phase where inflammatory responses, muscle inhibition, and altered biomechanics are present.

The use of surface electromyography (sEMG) to assess quadricep muscle activity provides valuable insights into neuromuscular adaptations during recovery [20]. Although sEMG has been widely used in TKA research, to the authors’ knowledge, no studies had examined its application in robot-assisted TKA, especially in conjunction with ROM measurements during tasks such as active knee extension and walking. Therefore, this study aimed to compare the operated and non-operated limbs four weeks after robot-assisted total knee arthroplasty in quadricep muscle activity during active knee extension and the 4-m walk test. Additionally, our objective was to compare the ROM of knee flexion–extension during the 4-m walk test between the limbs. We hypothesized that the operated limb would show a reduced range of motion and altered quadricep activation patterns compared to the non-operated limb at 4 weeks post surgery.

## 2. Methods

This cross-sectional observational study was conducted following the Strengthening the Reporting of Observational Studies in Epidemiology (STROBE) guidelines [21]. The study was approved by the European University of Madrid Research Committee (CIPI: 2024.899), and written informed consent was obtained from all participants before enrollment, adhering to the principles of the Declaration of Helsinki.

### 2.1. Participants

Patients who underwent unilateral TKA using the MAKO robotic-assisted system between May 2023 and October 2024 were considered for inclusion. Inclusion criteria encompassed individuals aged 60 to 80 years who had completed a standardized four-week postoperative rehabilitation program and were capable of walking unaided for at least 4 meters. Exclusion criteria included a history of bilateral TKA or previous knee surgeries, contralateral knee pain, presence of neurological or musculoskeletal conditions affecting lower limb function, and non-compliance with rehabilitation protocols or significant postoperative complications. Younger patients were excluded to reduce sample heterogeneity and avoid confounding differences in baseline function, recovery expectations, and activity levels associated with younger age groups.

### 2.2. Surgical Procedure

The TKA was performed using the MAKO robotic-arm-assisted system (Stryker, Kalamazoo, MI, USA), which integrates preoperative imaging with intraoperative execution for enhanced surgical precision. Preoperatively, patients underwent a computed tomography (CT) scan of the knee, allowing for the creation of a 3D model to guide implant positioning. The surgeon planned bone resections and implant placements tailored to the patient’s unique anatomy. During surgery, the robotic arm provided feedback to ensure precision, minimizing soft tissue damage and optimizing implant alignment and balancing [19].

### 2.3. Postoperative Rehabilitation Protocol

Following surgery, all patients participated in a standardized four-week rehabilitation program designed to restore knee function and facilitate a return to daily activities. The rehabilitation program aimed to address the common postoperative challenges of reduced ROM, muscle weakness, and impaired mobility (Table 1). All participants followed the same standardized rehabilitation program.

### 2.4. Outcome Measures

During the initial session, participants were familiarized with the experimental protocol to ensure proper execution of the tasks and reliable data collection. The session included a detailed explanation of the procedures, followed by practical familiarization with the two main tasks: the active knee extension test and the 4-m walking test. These tasks were designed to evaluate quadricep muscle activation in both static and dynamic conditions.

### 2.5. Quadriceps Muscle Activity

For the active knee extension task, participants were seated with their hips in 85 degrees and knees in 60 degrees of knee flexion and instructed to extend the knee to its maximum active range [11]. They were instructed to extend their knee to its maximum active range of motion while maintaining consistent posture. sEMG data were collected during three repetitions, and the mean activity across trials was calculated for analysis. For the 4-m walking test, participants walked at a self-selected pace along a flat, unobstructed path while sEMG signals were continuously recorded to capture muscle activation throughout the gait cycle [22].

sEMG was utilized to assess the activity of the vastus lateralis and vastus medialis muscles during active knee extension and the 4-m walking test. A BTS FreeEMG system (BTS Bioengineering, Milan, Italy) was employed for data acquisition; this is a wireless system known for its reliability and ease of application in dynamic tasks. The electrodes were placed following SENIAM guidelines [23]:

- Vastus Lateralis: Positioned longitudinally over the muscle belly, approximately two-thirds of the distance between the anterior superior iliac spine and the lateral border of the patella.
- Vastus Medialis: Positioned longitudinally over the muscle belly, approximately 4 cm proximal to the patella and 1–2 cm medial to the midline of the thigh [23,24].

Electrodes were placed on the skin after preparation to ensure optimal signal quality. Skin preparation involved shaving the electrode sites, abrading lightly with fine sandpaper to remove the superficial layer of skin, and cleaning the area with isopropyl alcohol to minimize impedance.

sEMG signals were acquired with a sampling frequency of 1000 Hz and amplified using the BTS system’s integrated amplifiers. The raw signals were band-pass filtered at 40–300 Hz to remove noise and motion artifacts while preserving the signal’s physiological components. The signals were then rectified and smoothed using a root mean square (RMS) algorithm with a 50 ms window to derive the signal envelope [25,26].

Outcome measures included maximum activity (µV), which corresponded to the peak activation recorded during each task.

### 2.6. Statistical Analysis

The normality of the data was assessed using the Kolmogorov–Smirnov test. Descriptive statistics were calculated for quantitative variables as means ± standard deviations (SDs) and for qualitative variables as frequencies and percentages. A linear mixed-effects model was employed to evaluate the effects of the operated side and gender on the outcomes. This model included operated side and gender as fixed factors, with their interaction also considered. Partial eta-squared (η^2^p) value was used to assess the effect size, with η^2^ values categorized as small (0.01), medium (0.06), or large (0.14). Pairwise comparisons were conducted as post hoc analyses using Bonferroni correction to control multiple comparisons. A significant level of 0.05 was used for all analyses. A post hoc power analysis revealed that the observed power for the linear mixed model exceeded 0.80, suggesting that the sample size was sufficient to detect the effects of interest. Statistical analyses were performed using SPSS v29 for Windows.

## 3. Results

The initial sample consisted of 213 participants, with 113 females and 100 males. After data filtering for EMG quality, 112 participants were excluded, resulting in a final sample of 101 participants (50 females and 51 males). These exclusions were based on predefined signal quality thresholds and were attributed to common issues such as movement artifacts, improper electrode adherence, and excessive skin impedance.

The average age of the final sample was 69.8 ± 8.3 years, with females being significantly older than males (*p* = 0.008). Males were significantly taller, with an average height compared to females (*p* < 0.001). Similarly, males had a higher average weight compared to females (*p* < 0.001). Despite these differences in height and weight, there was no significant difference in BMI between the sexes (*p* = 0.963). Regarding the affected side, the right side was involved in 56.8% of participants, with no significant differences between sexes (*p* = 0.783) (Table 2).

### 3.1. Knee Extension Test

Statistical analysis revealed significant differences in several variables between groups and conditions. In terms of muscle activity, the vastus lateralis during the knee extension test showed higher activation on the non-operated side compared to the operated side in males (F = 19.547, *p* < 0.001). Similarly, significant differences were observed for the vastus medialis during the knee extension test, with males showing higher activation on the non-operated side (F = 14.100, *p* < 0.001) (Table 3).

### 3.2. 4-m Walking Test

Additionally, during the 4-m walking test, the vastus lateralis displayed a significant difference between the operated and non-operated sides in both males and females (F = 8.943, *p* = 0.004). For range of motion, significant side effects were noted for the flexion–extension range during the 4-m test (F = 6.503, *p* = 0.012), indicating greater movement on the non-operated side. No significant differences were found in vastus medialis activity during the 4-m walking test or in the interaction effects of sex and side across all measured variables (*p* > 0.05) (Table 4).

### 3.3. Pairwise Comparisons

**Figure 1:**
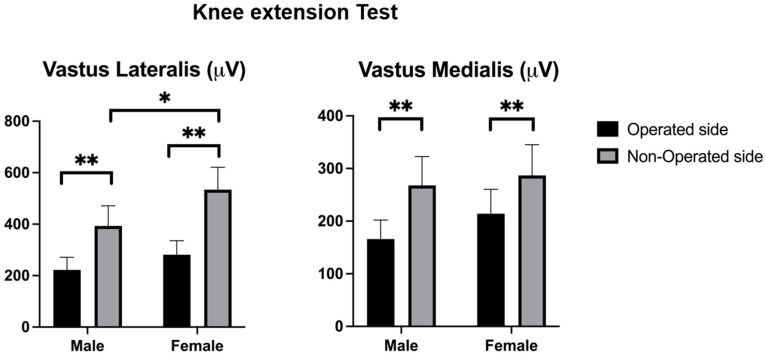
Quadriceps muscle activity during the knee extension test. Vastus lateralis and vastus medialis muscle activities (µV) during the knee extension test are shown. The black bars represent the operated side while the gray bars represent the non-operated side. Statistical significance: * *p* < 0.05, ** *p* < 0.01.

For the knee extension test, males exhibited significantly higher vastus lateralis activity on the non-operated side compared to the operated side with a mean difference of 174 µV (95% CI [90, 258], *p* < 0.001) while females also showed significantly higher activity on the non-operated side with a mean difference of 238 µV (95% CI [152, 324], *p* < 0.001) (Figure 1). Regarding vastus medialis activity, males demonstrated significantly higher activation on the non-operated side compared to the operated side, with a mean difference of 102 µV (95% CI [34, 169], *p* = 0.003), and females showed a similar pattern with significantly higher activity on the non-operated side with a mean difference of 137 µV (95% CI [47, 226], *p* = 0.003) (Figure 1).

**Figure 2:**
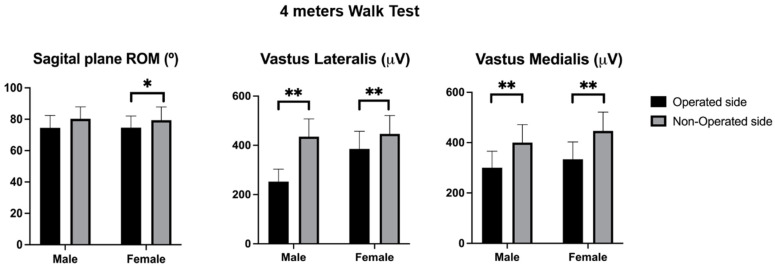
Range of motion and quadricep muscle activity during the 4-m walking test. Sagittal plane range of motion (°) and vastus lateralis and vastus medialis muscle activities (µV) during the 4-m walking test are shown. The black bars represent the operated side while the gray bars represent the non-operated side. Statistical significance: * *p* < 0.05, ** *p* < 0.01.

In the 4-m walking test, females exhibited a significantly greater range of motion (flexion–extension) on the non-operated side compared to the operated side, with a mean difference of 7.691° (95% CI [0.309, 15.073], *p* = 0.041), while no significant differences were observed in males (*p* > 0.05) (Figure 2). Males showed significantly higher vastus lateralis activity on the non-operated side compared to the operated side, with a mean difference of 171 µV (95% CI [70, 272], *p* = 0.001), and females demonstrated higher activity on the non-operated side with a mean difference of 151 µV (95% CI [47, 255], *p* = 0.005) (Figure 2). For the vastus medialis, males exhibited significantly higher activity on the non-operated side compared to the operated side, with a mean difference of 99 µV (95% CI [12, 185], *p* = 0.026), while females showed a similar trend with higher activity on the non-operated side, with a mean difference of 137 µV (95% CI [47, 226], *p* = 0.003) (Figure 2).

## 4. Discussion

Patients who underwent robot-assisted total knee arthroplasty exhibited significant asymmetries in quadricep activation and the ROM during early recovery. The vastus lateralis and vastus medialis muscles showed significantly lower activation on the operated side compared to the non-operated side in both the knee extension test and the 4-m walking test, with greater deficits observed in females. Females exhibited a significantly lower knee ROM on the operated side compared to the non-operated side whereas no significant ROM differences were found in males.

The findings of the present study were consistent with previous research reporting quadricep weakness following TKA. These results align with studies identifying arthrogenic muscle inhibition as a key factor contributing to quadricep weakness in the early recovery phase [11,16]. This inhibition is caused by postoperative inflammation and pain, leading to neuromuscular deficits and a decrease in voluntary muscle activation [27].

Our findings align with previous literature documenting neuromuscular deficits following conventional TKA, particularly in quadricep activation and the range of motion. For example, Mizner et al. reported a strength loss exceeding 60% in the quadriceps of the operated limb during the first month after traditional TKA, highlighting the early impact of surgery on muscle performance [15,16]. Similarly, Yoshida et al. observed persistent quadricep strength asymmetry between limbs up to one-year post surgery [17]. Compared to these studies, our results in a robot-assisted context showed comparable or slightly attenuated asymmetries in vastus lateralis and medialis activity at 4 weeks, suggesting that while robotic techniques may improve alignment and soft-tissue handling, early postoperative neuromuscular deficits remain present. This highlights the idea that surgical precision alone does not eliminate the need for targeted rehabilitation to address early motor impairments.

Evidence suggests that these deficits may persist for months or even years, affecting patient functionality and leading to gait alterations [17]. Despite advancements in surgical precision with robotic-assisted techniques, our findings confirm that early neuromuscular impairments persist. The benefits of robot-assisted TKA have been widely documented, demonstrating improved alignment and greater postoperative joint stability [8,13]. Some studies have even reported a reduced risk of joint stiffness and improved pain scores on the pain intensity in the immediate postoperative period, 24–72 h [19].

However, the present study was one of the first to analyze quadricep muscle activity in patients undergoing robot-assisted TKA, suggesting that despite the surgical precision provided by robotic technology, muscle activation deficits persist in the early postoperative phase. While further research is needed, it is possible that although alignment may influence long-term outcomes, it does not mitigate neuromuscular inhibition in the acute phase of the rehabilitation process.

### 4.1. Sex-Based Differences in Recovery

The results indicate that women may experience greater neuromuscular deficits following TKA, as evidenced by greater asymmetry in the quadricep ROM. Previous studies have reported that women tend to have a slower recovery after TKA. These findings may be attributed to preoperative patient status, including lower strength levels, increased pain sensitivity, reduced muscle mass, and impaired neuromuscular control [18].

Further research is needed to determine whether rehabilitation protocols should be sex-specific to optimize recovery outcomes. However, there is existing evidence on sex differences in muscle activation and functional recovery following knee injuries and surgeries [28]. Therefore, developing rehabilitation strategies tailored to these differences appears to be a promising approach.

### 4.2. Clinical Implications

The significant reduction in quadricep activation on the operated side highlights the critical need for neuromuscular rehabilitation strategies aimed at restoring muscle function. The early optimization of rehabilitation protocols is essential as persistent neuromuscular inhibition may increase the risk of falls and compromise functional recovery. Interventions focusing on early muscle activation and strength restoration could mitigate these risks and support a faster return to daily activities.

The observed asymmetry in the ROM during gait underscores the importance of incorporating gait retraining to prevent compensatory movement patterns that could contribute to long-term functional impairments. Progressive rehabilitation programs emphasizing quadricep strengthening are crucial as they enhance functionality without compromising the ROM. A comprehensive approach that integrates both strength and gait training may facilitate symmetrical recovery and reduce the likelihood of chronic deficits.

Beyond the ROM, assessing strength and functional performance is vital for providing a holistic understanding of postoperative recovery. These outcomes serve as key indicators of rehabilitation efficacy and their relationship with surgical outcomes. Therefore, integrating functional assessments into postoperative care can guide treatment adjustments and improve patient outcomes.

The findings suggest that implementing early postoperative interventions, such as NMES and task-specific strength training, may help mitigate persistent neuromuscular inhibition and accelerate functional recovery. NMES, combined with task-oriented exercises, has demonstrated efficacy in enhancing quadricep activation, especially in the early postoperative phase. Future research should explore the differential impact of various rehabilitation strategies on quadricep activation and functional recovery following robot-assisted TKA. Specifically, longitudinal studies are essential to determine whether early postoperative muscle deficits contribute to long-term functional limitations and disability.

Additionally, given the observed sex-based differences in recovery patterns, future studies should investigate whether rehabilitation protocols tailored to patient sex can enhance outcomes. Personalized approaches considering sex-specific neuromuscular adaptations may optimize recovery and address disparities in postoperative outcomes [13].

### 4.3. Limitations

However, the study was limited by its cross-sectional design, which prevented the evaluation of long-term recovery trajectories. Future research should incorporate longitudinal follow-ups to determine whether these asymmetries persist or become resolved over time. Longitudinal studies are needed to evaluate whether the early improvements in mobility and muscle activation observed with robotic TKA are sustained over time. Follow-up assessments at 6 months, 1 year, and beyond 5 years would provide critical data on the long-term benefits and durability of robotic-assisted interventions. Additionally, pain intensity and the presence of comorbidities, which may influence postoperative recovery, were not included in the present analysis. Pain was not measured as the primary focus of this study was on the objective assessments of muscle activation and gait-related kinematics. Similarly, although comorbidities were documented during participant screening, no subgroup comparisons were performed. These factors should be considered in future studies to provide a more comprehensive view of postoperative recovery in TKA patients. Although the sample used in this study was representative, future research with larger cohorts could enhance the generalizability of the findings. Moreover, the use of surface-electromyography sEMG has inherent limitations. sEMG is susceptible to signal contamination from crosstalk between adjacent muscles, variability in electrode placement, and changes in skin impedance, which may affect the accuracy of muscle activation measurements. Additionally, sEMG captures electrical activity rather than actual muscle force, which limits the interpretation of neuromuscular performance. It is also important to note that, as an observational study, the results reflect associations rather than causal relationships. The observed differences in muscle activation and range of motion cannot be directly attributed to robot-assisted TKA without considering other potential contributing factors. Experimental designs or randomized controlled trials are needed to establish causality. Future research could explore how robot-assisted total knee arthroplasty specifically influences the activation of different lower limb muscle groups—including the quadriceps, hamstrings, and gastrocnemius—during the early phases of recovery. Finally, the study did not assess functional outcomes beyond sEMG measurements and the ROM, such as gait analysis or patient-reported outcomes, which could provide a more comprehensive understanding of recovery following robot-assisted TKA. These limitations should be considered when interpreting the findings, and future research should aim to address these gaps to advance the understanding of postoperative recovery in TKA patients.

## 5. Conclusions

This study highlighted significant asymmetries in quadricep-muscle sEMG and the ROM following robot-assisted TKA at the 4-week follow-up, with more pronounced deficits observed in women. These results underscore the need for personalized, sex-specific rehabilitation interventions, particularly during the early postoperative phase, to promote muscle reactivation and optimize functional recovery.

## Figures and Tables

**Table 1:** Rehabilitation interventions following total knee arthroplasty (TKA).

Intervention	Description
Range of Motion (ROM) Exercises	Passive and active-assisted exercises to improve knee flexion and extension, aiming to achieve at least 0° to 90° of motion within the first two weeks.
Strength Training	Strengthening the quadriceps, hamstrings, and hip muscles through isometric and isotonic exercises, progressing from closed-chain to open-chain activities as tolerated.
Neuromuscular Electrical Stimulation (NMES)	NMES was applied to the quadricep muscle to enhance muscle activation and strength.
Gait Training	Patients practiced weight-bearing and ambulation exercises, progressing from the use of assistive devices to independent walking.

**Table 2:** Descriptive analyses of the sample.

Variables	Sample	Females (n = 50)	Males (n = 51)	Difference
Age, years	69.8 ± 8.3	71.2 ± 6.9	68.2 ± 9.4	3.0 (0.8;5.2) *p* = 0.008
Height, meters	1.67 ± 0.08	1.62 ± 0.06	1.73 ± 0.07	0.1 (0.09;0.12) *p* < 0.001
Weight, kilograms	80.0 ± 15.4	75.1 ± 15.3	85.6 ± 13.6	10.5 (6.6;14.4) *p* < 0.001
BMI, kg/m^2^	28.5 ± 4.8	28.5 ± 5.6	28.5 ± 3.7	0.0 (−1.3;1.3) *p* = 0.963
Right side affected, n (%)	60 (56.8)	63 (55.8)	58 (58.0)	*p* = 0.783

**Table 3:** Range of movement and quadricep activity differences during the knee extension test.

Variables	Operated Side (n = 101)		Non-Operated Side (n = 101)		Inter-Subjects Effects (F; *p*-Value (η^2^p))		
	Female (n = 50)	Male (n = 51)	Female (n = 50)	Male (n = 51)	Sex	Side	Sex*Side
Peak Muscle Activity (µV)							
Vastus lateralis	281.2 ± 192.6	221.2 ± 175.6	534.1 ± 307.4	393.1 ± 278.8	F = 4.814; *p* = 0.031 (0.05)	F = 46.104; *p* < 0.001 (0.32)	F = 1.118; *p* = 0.293 (0.01)
Vastus medialis	214.3 ± 162.2	166.0 ± 128.7	286.7 ± 204.9	267.5 ± 194.9	F = 1.462; *p* = 0.229 (0.05)	F = 13.348; *p* < 0.001 (0.12)	F = 0.270; *p* = 0.604 (0.00)

**Table 4:** Range of movement and quadricep activity scores during the 4-m walking test.

Variables	Operated Side (n = 101)		Non-Operated Side (n = 101)		Inter-Subjects Effects (F; *p*-Value (η^2^p))		
	Female (n = 50)	Male (n = 51)	Female (n = 50)	Male (n = 51)	Sex	Side	Sex*Side
Range of movement (degrees)							
Flexion–extension	74.6 ± 26.0	74.5 ± 28.1	79.4 ± 29.5	80.3 ± 26.8	F = 0.015; *p* = 0.903 (0.00)	F = 6.503; *p* = 0.012 (0.063)	F = 0.175; *p* = 0.677 (0.00)
Peak Muscle activity (µV)							
Vastus lateralis	385.5 ± 252.6	252.5 ± 181.7	492.4 ± 298.5	435.2 ± 258.2	F = 8.943; *p* = 0.004 (0.084)	F = 19.547; *p* < 0.001 (0.17)	F = 0.075; *p* = 0.784 (0.00)
Vastus medialis	333.5 ± 243.9	300.5 ± 232.3	446.5 ± 262.5	400.1 ± 254.5	F = 1.011; *p* = 0.317 (0.01)	F = 14.100; *p* < 0.001 (0.13)	F = 0.366; *p* = 0.547 (0.00)

## Data Availability

Data (raw) are available upon formal request from the corresponding author.
